# Aggresome formation and liquid–liquid phase separation independently induce cytoplasmic aggregation of TAR DNA-binding protein 43

**DOI:** 10.1038/s41419-020-03116-2

**Published:** 2020-10-23

**Authors:** Seiji Watanabe, Hidekazu Inami, Kotaro Oiwa, Yuri Murata, Shohei Sakai, Okiru Komine, Akira Sobue, Yohei Iguchi, Masahisa Katsuno, Koji Yamanaka

**Affiliations:** 1 Department of Neuroscience and Pathobiology, Research Institute of Environmental Medicine, Nagoya University, Nagoya, Aichi 464-8601 Japan; 2 Department of Neuroscience and Pathobiology, Nagoya University Graduate School of Medicine, Nagoya, Aichi 466-8550 Japan; 3 Department of Neurology, Nagoya University Graduate School of Medicine, Nagoya, Aichi 466-8550 Japan

**Keywords:** Mechanisms of disease, Amyotrophic lateral sclerosis

## Abstract

Cytoplasmic inclusion of TAR DNA-binding protein 43 (TDP-43) is a pathological hallmark of amyotrophic lateral sclerosis (ALS) and a subtype of frontotemporal lobar degeneration (FTLD). Recent studies have suggested that the formation of cytoplasmic TDP-43 aggregates is dependent on a liquid–liquid phase separation (LLPS) mechanism. However, it is unclear whether TDP-43 pathology is induced through a single intracellular mechanism such as LLPS. To identify intracellular mechanisms responsible for TDP-43 aggregation, we established a TDP-43 aggregation screening system using a cultured neuronal cell line stably expressing EGFP-fused TDP-43 and a mammalian expression library of the inherited ALS/FTLD causative genes, and performed a screening. We found that microtubule-related proteins (MRPs) and RNA-binding proteins (RBPs) co-aggregated with TDP-43. MRPs and RBPs sequestered TDP-43 into the cytoplasmic aggregates through distinct mechanisms, such as microtubules and LLPS, respectively. The MRPs-induced TDP-43 aggregates were co-localized with aggresomal markers and dependent on histone deacetylase 6 (HDAC6), suggesting that aggresome formation induced the co-aggregation. However, the MRPs-induced aggregates were not affected by 1,6-hexanediol, an LLPS inhibitor. On the other hand, the RBPs-induced TDP-43 aggregates were sensitive to 1,6-hexanediol, but not dependent on microtubules or HDAC6. In sporadic ALS patients, approximately half of skein-like TDP-43 inclusions were co-localized with HDAC6, but round and granular type inclusion were not. Moreover, HDAC6-positive and HDAC6-negative inclusions were found in the same ALS patient, suggesting that the two distinct pathways are both involved in TDP-43 pathology. Our findings suggest that at least two distinct pathways (i.e., aggresome formation and LLPS) are involved in inducing the TDP-43 pathologies.

## Introduction

Abnormal accumulation of TAR DNA-binding protein-43 (TDP-43) is a pathological hallmark of amyotrophic lateral sclerosis (ALS), a fatal neurodegenerative disease characterized by a selective loss of motor neurons, and a subtype of frontotemporal lobar degeneration (FTLD-TDP)^1^. TDP-43 is a DNA/RNA-binding protein that is predominantly localized in nuclei and plays multifunctional roles in RNA metabolism, including pre-mRNA splicing, translational control, and mRNA stability^2,3^. However, in most of the ALS and FTLD-TDP cases, TDP-43 is mislocalized into the cytoplasm and forms inclusion bodies^4,5^. In addition to this, more than 50 mutations in the *TARDBP* gene, encoding TDP-43, have been identified as a cause of inherited ALS^6^. These observations suggest that dysfunction of TDP-43 is a significant component of ALS pathogenesis. Thus, understanding the mechanism of TDP-43 aggregation will uncover the mechanistic basis for TDP-43 pathology and neurodegeneration in ALS/FTLD.

Recently, a number of studies revealed that many kinds of RNA-binding proteins (RBPs), including TDP-43, spontaneously develop granule-like structures via a liquid–liquid phase separation (LLPS) mechanism^7,8^. LLPS is a process in which proteins and nucleotides abruptly segregate into two distinct phases, enabling the formation of intracellular membrane-less organelles^9^, such as p-bodies^10^ and stress granules^11,12^. Under physiological conditions, LLPS enables to achieve high local concentrations for molecular interactions and rapid chemical reactions, and allow fast changes of molecules upon signaling for facilitating various intracellular biological processes, e.g., transcriptional regulation and signal transduction^13^. However, once excess amounts of proteins are accumulated together with dysregulation of LLPS, the complexes quickly transform into pathological inclusions that are often found in neurodegenerative diseases^14,15^. ALS causative gene products, including FUS, TIA-1, and, of course, TDP-43, are proposed to form aggregates via LLPS. Consistent with this hypothesis, recent studies have discovered that optical multimerization of cytoplasmic TDP-43 induces the aggregation and sequestration of endogenous nuclear TDP-43 into the cytoplasmic aggregates that are dependent on LLPS^16,17^.

However, whether LLPS is solely responsible for the cytoplasmic aggregation of TDP-43 remains unclear. Among the over 25 inherited ALS/FTLD causative genes, there is a considerable number of the genes encoding the proteins that have the functions different from RNA regulation^18,19^. For instance, *SQSTM1* and *TBK1* encode proteins involved in autophagy, while *PFN1* and *TUBA4A* encode cytoskeletal proteins. Considering that TDP-43 pathology is also observed in the most of inherited ALS/FTLD patients, there may be the intracellular mechanisms other than LLPS stimulating the formation of TDP-43 aggregates in ALS/FTLD lesions.

In this study, we aimed to determine the intracellular pathways involved in the formation of TDP-43 aggregates. Using a screening system of cytoplasmic TDP-43 aggregations induced by the expression of inherited ALS/FTLD causative genes, we established that, in addition to the RBPs, microtubule-related proteins (MRPs) also co-aggregated with TDP-43 in the cytoplasm. The MRPs induced co-aggregation of TDP-43 via the formation of aggresomes, cytoplasmic aggregations that are dependent on microtubules. In contrast, the RBPs-induced aggregations were dependent on LLPS. Therefore, both the TDP-43 co-aggregation pathways were independent from each other. Moreover, we also showed that a subset of TDP-43 inclusions in spinal motor neurons of sporadic ALS cases were co-localized with histone deacetylase 6 (HDAC6), an aggresome marker. Our findings suggest that at least two distinct pathways (i.e., aggresome formation and LLPS) are involved in inducing the TDP-43 pathologies.

## Materials and methods

### Antibodies

Antibodies used in this study are as follows; anti-HDAC6 (1:1000 for immunoblotting, 1:50 for immunohistochemistry, #7558, RRID: AB_10891804, Cell Signaling Technology, Danvers, MA, USA), anti-γ-tubulin (1:1000, #ab27074, RRID: AB_2211240, Abcam, Cambridge, UK), anti-vimentin (1:50, #3932, RRID: AB_2288553, Cell Signaling), anti-TDP-43 (1:1000, clone 3H8, #MABN45, EMD Millipore, Billerica, MA), anti-β-actin (1:5000, #5441, RRID: AB_476744, Sigma-Aldrich Co LLC, St. Louis, MO,USA), anti-RFP (1:1000, #PM005, RRID: AB_591279, Medical & Biological Laboratories (MBL) Co LTD, Nagoya, Japan).

### Cell culture and small-interfering RNA (siRNA) transfection

Mouse neuroblastoma Neuro2a (RRID: CVCL_0470) (N2a) cells were maintained and differentiated, as described elsewhere^20^. To establish the cell line stably expressing EGFP-fused human TDP-43 wild-type or nuclear localization signal (NLS)-deficient mutant^21^, we transfected linearized pcDNA3.1(+) (Thermo Fisher Scientific Inc, Waltham, MA, USA) inserted each TDP-43 complementary DNA (cDNA) with Lipofectamine 2000 (Thermo Fisher). The cells were cultured for two weeks in a growth medium containing 1.0 g/L G-418 (Nacalai Tesque, Kyoto, Japan). The colonies were isolated and confirmed the expression of EGFP-TDP-43 by fluorescent microscopy and immunoblotting. The established cell lines were maintained in a growth medium containing 1.0 g/L G-418. HeLa (CCL-2) and HEK293 (CRL-1573) cells, both obtained from ATCC, were maintained in Dulbecco’s Modified Eagle’s’ Medium (DMEM) containing 4.5 g/L glucose supplemented with 10% (v/v) fetal bovine serum (FBS), 100 U/mL penicillin, and 100 µg/mL streptomycin (all from Thermo Fisher) at 37 °C in a humidified chamber containing 5% CO_2_. Total RNAs of HeLa and HEK293 cells were isolated using the RNeasy-micro kit (QIAGEN, Hilden, Germany).

Stealth siRNA against the murine *Hdac6* gene was obtained from Thermo Fisher. siRNA (20 nM) was transfected into N2a cells using Lipofectamine RNAi Max (Thermo Fisher) as described by the manufacturer.

### TDP-43 aggregation screening using mammalian inherited ALS/FTLD causative gene expression library

Full-length wild-type cDNA of each familial ALS causative gene was amplified using the gene-specific primers from cDNA synthesized from total RNA of HeLa or HEK293 cells. The amplified cDNA was inserted into pmCherry-N1/C1 vectors (Clontech Laboratories Inc, Mountain View, CA, USA) using SLiCE reaction^22^. ALS/FTLD-causing mutations were introduced according to the guidance of the Primestar Mutagenesis Basal Kit (Takara Bio, Shiga, Japan).

N2a cells stably expressing EGFP-fused TDP-43, which is described above, were seeded at 5.0 × 10^4^ /well on a 4 well cover-slide chamber (AGC Techno Glass Inc, Shizuoka, Japan) coated with poly-D-lysine. On the next day, 0.25 µg/well plasmids to express inherited ALS/FTLD causative genes were transfected with Lipofectamine 2000 (Thermo Fisher) according to the manufacturer’s guide. After 6 h of the transfection, the medium was changed into a differentiation medium (DMEM + 2%(v/v) fetal bovine serum and 2 mM N6,2′-O-dibutyryladenosine-3′,5′-cyclic monophosphate (Nacalai Tesque, Kyoto, Japan)), and cultured for 48 h. Then, the non-fixed cells were observed using laser scanning confocal microscopy (LSM-700; Carl Zeiss AG, Oberkochen, Germany) and the equipped software (Zen; Carl Zeiss AG).

For the treatment with 1,6-hexanediol (1,6-HD) (Sigma), the medium was replaced with a fresh one with/without 4%(v/v) 1,6-HD and incubated for 10 min at room temperature. Then, the non-fixed cells were observed using laser scanning confocal microscopy.

### Immunoblotting and Immunofluorescence

Immunoblotting analyses were performed as described elsewhere^23^. For TDP-43 insolubility assay, the cells were first lysed in TBS supplemented with 1 mM EDTA, 1%(v/v) Triton X-100 and protease inhibitor cocktail by brief sonication on ice. After centrifugation at 15,000 × *g*, at 4 °C for 5 min, the supernatants were collected as soluble fraction. The pellets were resuspended in TBS supplemented with 1 mM EDTA and 2%(w/v) sodium-dodecyl sulfate (SDS) by brief sonication, then they were subjected to centrifugation at 15,000 × *g*, room temperature for 5 min. The supernatants were collected as insoluble fractions. Aliquots containing 15 µg/lane of soluble fractions and the same volume of corresponding insoluble fractions were analyzed by immunoblotting. For immunofluorescence analyses of γ-tubulin and vimentin, cultured N2a cells were transfected and cultured in a differentiation medium for 48 h. Then, the cells were fixed with ice-cold methanol at −30 °C for 20 min. After three times wash with TBS (50 mM Tris-HCl (pH 7.4), 150 mM NaCl), the cells were immunostained as described previously^23^. Briefly, the cells were incubated with primary antibodies at 4 °C for over-night, following incubation with secondary antibodies at room temperature for 2 h. Immunofluorescence images were obtained by a confocal laser scanning microscopy (LSM-700; Carl Zeiss AG, Oberkochen, Germany) and the equipped software (Zen; Carl Zeiss AG).

### Postmortem human tissues

Specimens of spinal cords from six patients with sporadic ALS and two control patients with other neurological diseases were obtained by autopsy with informed consent (Supplementary Table S1). The diagnosis of ALS was confirmed by El Escorial diagnostic criteria as defined by the World Federation of Neurology. The ethics committee approved the collection of tissues and their use in this study of Nagoya University. We confirmed that informed consent of all the tested subjects were obtained. For immunofluorescence analyses, the sections were prepared from formalin-fixed and paraffin-embedded tissues, deparaffinized, and incubated at 90 °C for 40 min in HistVT One (Nacalai Tesque).

### Statistical analyses

All the data from immunofluorescence and semi-quantitative immunoblotting were analyzed by an unpaired *t*-test for the comparison between two groups, or one-way ANOVA followed by the posthoc Tukey’s multiple comparison *t*-test for the comparison among more than three groups, respectively. When the values were compared only with the control values, Dunnet’s multiple comparison tests were used instead of Tukey’s one. For all the ANOVA, we used Brown–Forsythe and Welch’s correction not to assume normal distribution of the data. All the statistical analyzes were carried out using GraphPad Prism software (GraphPad Software, La Jolla, CA).

## Results

### TDP-43 co-aggregation screening reveals the involvement of microtubule-related proteins (MRPs) and RNA-binding proteins (RBPs) in cytoplasmic TDP-43 aggregation

**Fig. 1:**
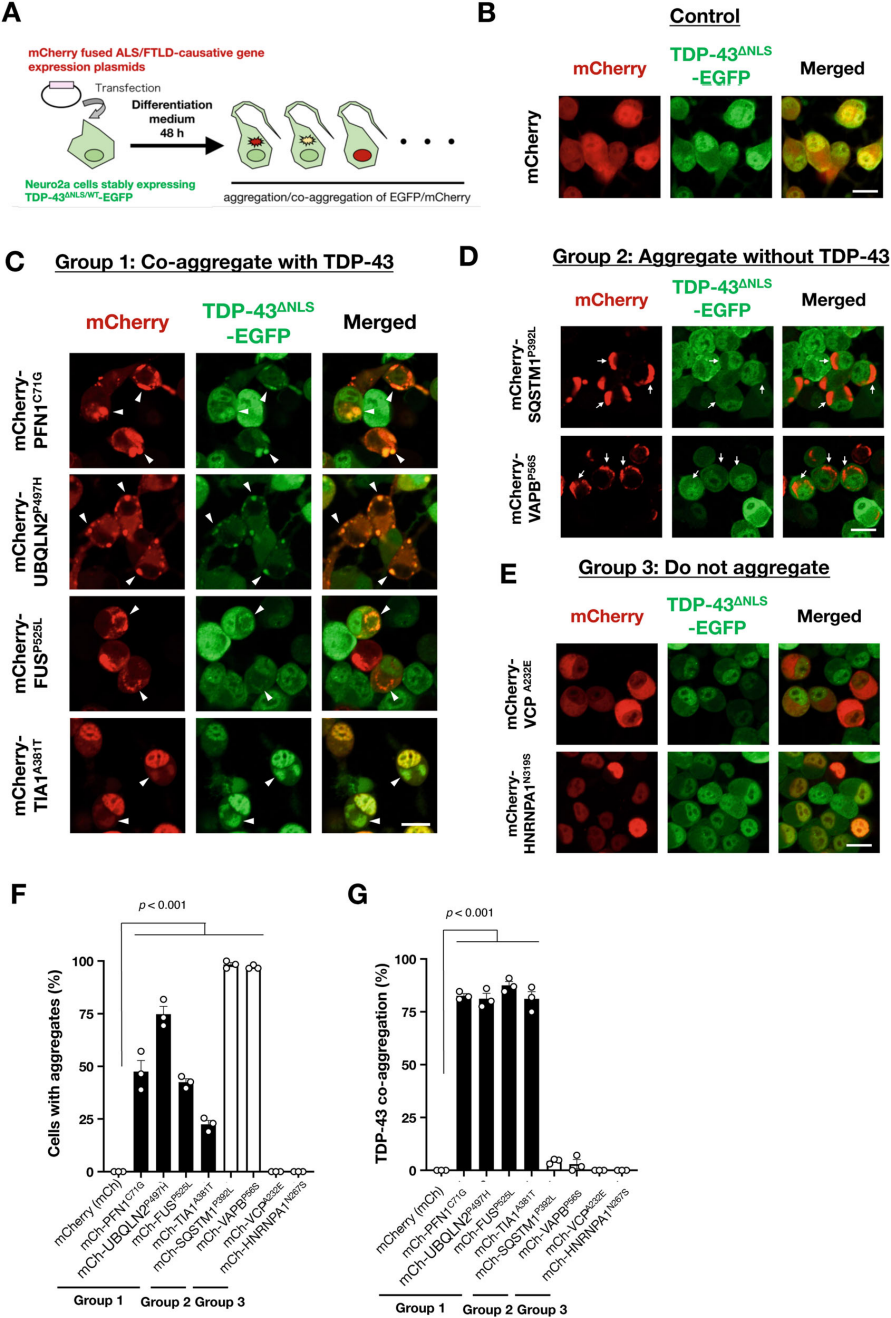
TDP-43 co-aggregation screening using a familial ALS/FTLD causative gene expression library. **A** Schematic illustration of the TDP-43 co-aggregation screening. **B**–**E** Representative images of the screening results with Neuro2a (N2a) cells with stably expressing EGFP-fused cytoplasmic TDP-43 mutants (TDP-43^∆NLS^-EGFP). The image expressing a control plasmid is shown in **B**. A total of 19 ALS/FTLD causative genes were classified into three groups: Group 1, genes that induce co-aggregates with TDP-43^∆NLS^ (**C**); Group 2, genes that induce aggregates without TDP-43^∆NLS^ (**D**); and Group 3, genes that do not form aggregates in the cells (**E**). The entire screening results are shown in Supplementary Fig. S1. Arrowheads in **C** indicate co-aggregates of the transfected proteins with TDP-43. Arrows in **D** indicate aggregates of the transfected proteins excluding TDP-43. Scale bars = 20 µm. **F**, **G** Quantification of N2a cells with aggregates of ALS/FTLD causative gene products (**F**) and the ratio of co-aggregation with TDP-43 (**G**). Data are expressed as mean ± standard error of the means (SEM) (*n* = 3). More than 50 cells were counted for the quantification.

To identify intracellular pathways involved in cytoplasmic TDP-43 aggregation, we established a screening system for the co-aggregation of TDP-43 and ALS/FTLD causative proteins (Fig. 1A). We generated N2a cells that stably expressed EGFP-fused wild-type or NLS-defective TDP-43 (TDP-43^WT^ or TDP-43^∆NLS^, respectively) as reporter cell lines to visualize TDP-43 aggregates (Fig. 1B). When both the cells were cultured in normal conditions, no TDP-43 aggregates were observed. We also prepared an inherited ALS/FTLD causative gene expression library (19 genes in total, see Table 1). The library consisted of expression plasmids of mCherry-fused wild-type or two independent variants carrying ALS/FTLD-linked mutations for each of the genes. Then, we transfected each plasmid into the reporter cells and observed them after two days of incubation. We then screened the library to identify the ALS/FTLD genes that induce cytoplasmic TDP-43 aggregation in N2a reporter cells that stably express EGFP-TDP-43^∆NLS^. The screening results are summarized in Table 1. Our screening identified that 14/19 (74%) gene products formed intracellular aggregates and 9 of them (9/19 in total, 47%) co-aggregated with TDP-43^∆NLS^. Representative images for the cells expressing each of the inherited ALS/FTLD causative genes are shown in Supplementary Fig. S1. We also found that the ALS/FTLD causative gene products are classified into three groups: gene products that induce co-aggregates with TDP-43 (Group 1) (Fig. 1C, F, G), gene products that induce aggregates without TDP-43 (Group 2) (Fig. 1D, F, G), and gene products that do not form aggregates (Group 3) (Fig. 1E–G).

**Table 1:** Results of TDP-43 co-aggregation screening with ALS/FTLD causative genes.

Functions	Gene symbol	Mutations	Localization	Aggregation	Co-aggregation with TDP-43	TDP-43 inclusions in inherited ALS cases	Co-localization with TDP-43 inclusions	References
RNA-binding proteins	*TARDBP*	Wild-type	Nuclear	−	−	Yes^43–45^	−	1. Pamphlett et al.^43^ 2. Van Deerlin et al.^44^ 3. Yokoseki et al.^45^
	(ALS10)	M337V	Nuclear	−	−			
		G348C	Nuclear	−	−			
		∆NLS	Nuclear	−	−			
	*FUS*	Wild-type	Nuclear	−	−	No^46,47^	− (Yes in sporadic ALS^32^)	1. Kwiatkowski et al.^46^ 2. Vance et al.^47^ 3. Ikenaka et al.^32^
	(ALS6)	R521C	Nuclear	−	−			
		P525L	Cytosol	++	++			
	*HNRNPA1*	Wild-type	Nuclear	−	−	Yes	Yes	1. Kim et al.^48^
	(ALS20)	D314V	Nuclear	−	−			
		N319S	Nuclear	−	−			
	*MATR3*	Wild-type	Nuclear	−	−	Yes^49,50^	Yes^49^^,^^50^	1. Johnson et al.^49^ 2. Tada et al.^50^
	(ALS21)	S85C	Nuclear	−	−			
		F115C	Nuclear + cytosol	+	+			
	*TAF15*	Wild-type	Nuclear + cytosol	+	++	Not reported	−	
		G391E	Nuclear + cytosol	+	++			
		R408C	Nuclear + cytosol	+	+			
	*TIA1*	Wild-type	Nuclear + cytosol	+	++	Yes^34,35^	Yes^34^/No^35^	1. Hirsch-Reinshagen et al.^34^ 2. Mackenzie et al.^25^
		P362L	Nuclear + cytosol	+	+			
		A381T	Nuclear + cytosol	+	+			
Intracellular trafficking	*FIG4*	Wild-type	Cytosol	+	−	Not reported	−	
	(ALS11)	Q403X	Cytosol	+	−			
		V654A	Cytosol	+	−			
	*CHMP2B*	wild-type	Cytosol	+	+	No	−	1. Cairns et al.^51^
	(ALS17)	I29V	Cytosol	+	+			
		Q206H	Nuclear + cytosol	+	+			
	*TFG*	Wild-type	Cytosol	+++	−	Not reported	−	
		G269V	Cytosol	+++	−			
		P285L	Cytosol	+++	−			
Protein degradation	*VCP*	Wild-type	Cytosol	−	−	Yes^51–53^	Not reporter (Yes in cultured cells^53^)	1. Cairns et al.^51^ 2. Neumann et al.^52^ 3. Gitcho et al.^53^
	(ALS14)	R155H	Cytosol	−	−			
		A232E	Cytosol	−	−			
	*UBQLN2*	Wild-type	Cytosol	+++	++	Yes^54–56^	Yes^56^ (Yes in cultured cells^54^)	1. Deng et al.^54^ 2. Williams et al.^55^ 3. Fahed et al.^56^
	(ALS15)	P497H	Cytosol	+++	++			
		P506T	Cytosol	+++	++			
	*SQSTM1*	Wild-type	Cytosol	++	−	Yes	Yes	1. Kovacs et al.^57^
	(FTLD-ALS3)	P392L	Cytosol	++	−			
		G425R	Cytosol	++	−			
	*TBK1*	Wild-type	Cytosol	−	−	Yes^58,59^	Not reported	1. Freischmidt et al.^58^ 2. Gijselinck et al.^59^
	(FTLS-ALS4)	K401E	Cytosol	−	−			
		∆690–713	Cytosol	−	−			
	*CCNF*	Wild-type	Nuclear	−	−	Not reported	−	
		S509P	Nuclear	−	−			
		S621G	Nuclear	−	−			
Cytoskelton	*PFN1*	Wild-type	Cytosol	−	−	Yes^60,61^	Not reported (Yes in cultured cells^61^)	1. Wu et al.^60^ 2. Smith et al.^61^
	(ALS18)	C71G	Cytosol	++	++			
		M114T	cytosol	−	−			
	*TUBA4A*	Wild-type	Cytosol	++	++	Not reported	−	
	(ALS22)	R320H	Cytosol	++	++			
		W407X	Cytosol	++	++			
ER stress response	*VAPB*	Wild-type	er	−	−	Not reported	−	
	(ALS8)	T46I	er	+	−			
		P56S	er	++	−			
Growth hormone receptor	*ERBB4*	Wild-type	er + cell surface	−	−	Not reported	−	
	(ALS19)	R927Q	er + cell surface	−	−			
		R1275W	er + cell surface	−	−			
Mitochondrial homeostasis	*CHCHD10*	Wild-type	Mito	−	−	Not reported	−	
	(FTLD-ALS2)	P34S	Mito	−	−			
		S59L	Mito	−	−			

“Aggregation” was evaluated by ratio with intracellar aggregates of each gene product. +: mild (<30%), ++: medium (30–70%), +++: severe (>70%).

“Co-aggregation with TDP-43” was evaluated co-localization of TDP-43 aggregates and aggregates of each gene product. +: partially, ++: completely.

More than 50 cells were counted in each trial (*n* = 3 each).

*er* endoplasmic reticullum, *mito* mitochondria.

**Fig. 2:**
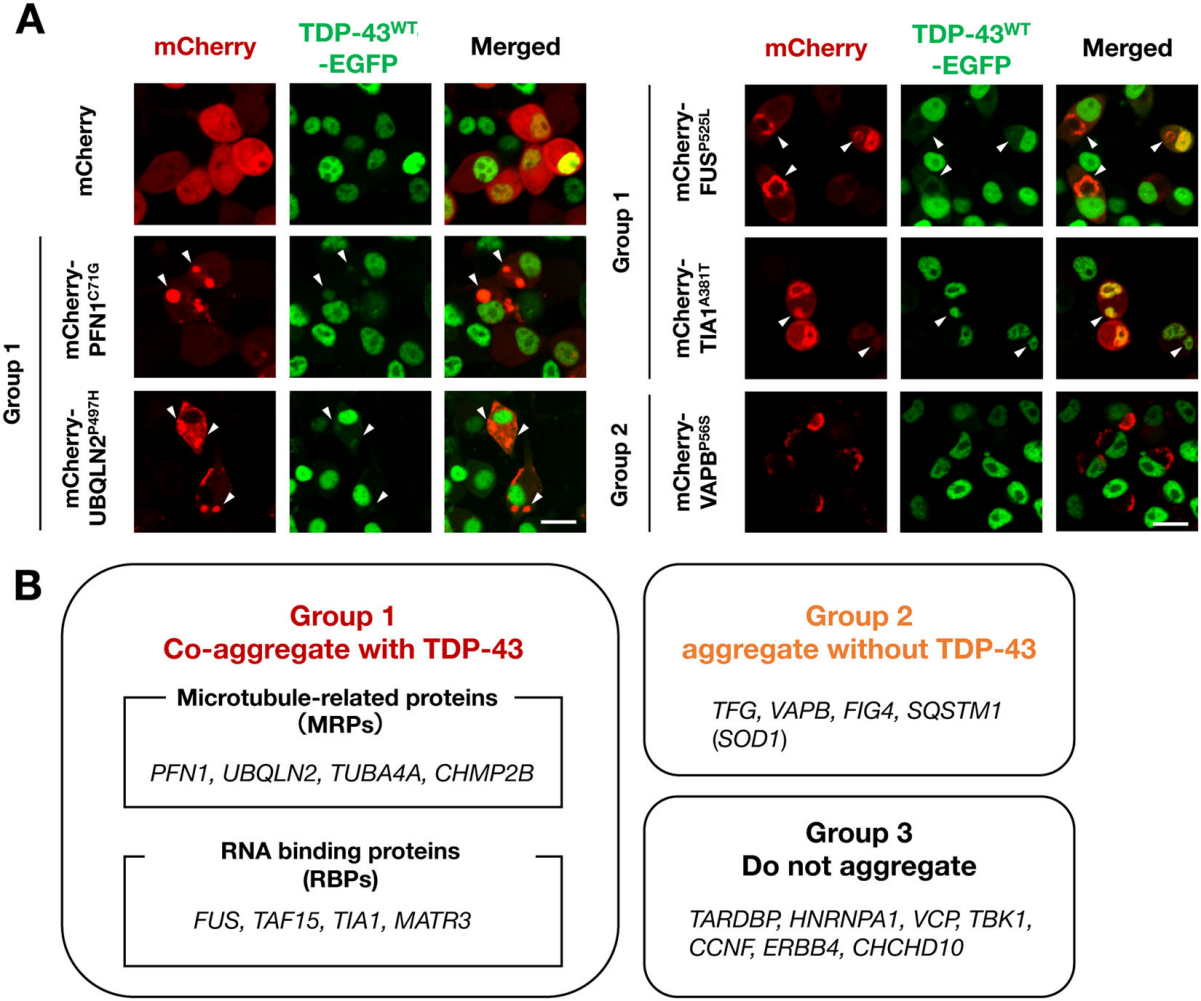
The ALS/FTLD causative genes co-aggregated with TDP-43 induce cytoplasmic translocation of wild-type TDP-43. **A** Representative images of the screening results with N2a cells stably expressing EGFP-fused wild-type TDP-43 (TDP-43^WT^). Arrowheads indicate cytoplasmic co-aggregates of the Group 1 gene products and TDP-43^WT^. Scale bars = 20 µm. **B** The summary of the TDP-43 co-aggregation screening. The genes in Group 1 were further classified into two subgroups based on their physiological functions: microtubule-related proteins (MRPs) and RNA-binding proteins (RBPs).

More importantly, all of the gene products in Group 1 induced cytoplasmic translocation and aggregation of TDP-43^WT^ (Fig. 2A and Supplementary Fig. S2). Interestingly, the Group 1 genes were involved in either microtubular dynamics or RNA regulation, suggesting that these two pathways are both involved in intracellular TDP-43 aggregation. Therefore, to determine the mechanisms of intracellular TDP-43 aggregation, we further classified Group 1 genes into microtubule-related proteins (MRPs; PFN1, UBQLN2, TUBA4A, and CHMP2B) and RNA-binding proteins (RBPs; FUS, TAF15, TIA1, and MART3) (Fig. 2B). Notably, the genes encoding RBPs-induced TDP-43 co-aggregation only when their mutants were mislocalized into the cytoplasm, suggesting that leakage of RBPs from nuclei is closely associated with the cytoplasmic TDP-43 aggregation.

**Fig. 3:**
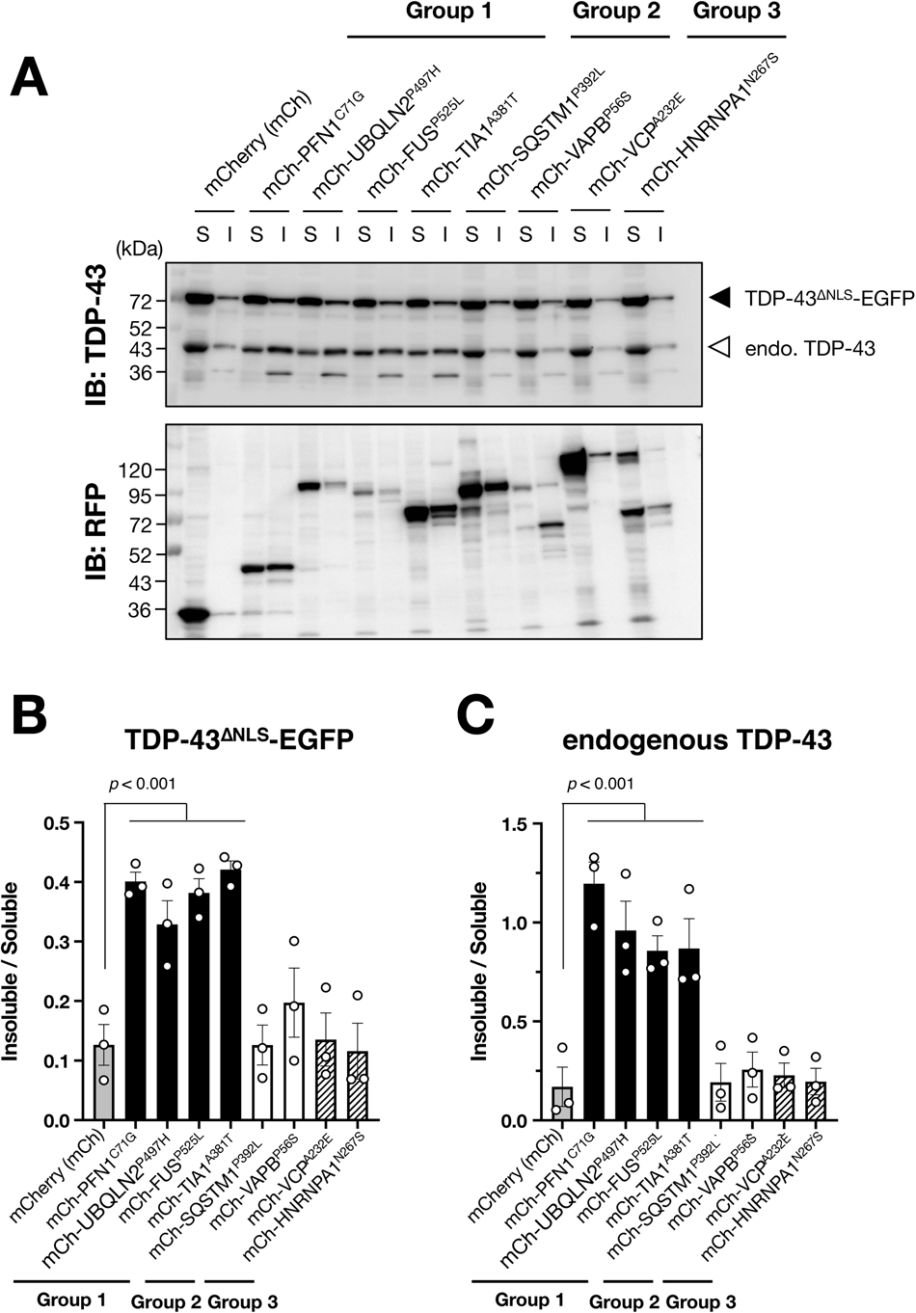
RBPs and MRPs decrease solubility of TDP-43. **A** Representative immunoblotting images showed the expressions of TDP-43 and mCherry (RFP) in soluble (S) or insoluble (I) fractions of N2a cells stably expressing TDP-43^∆NLS^-EGFP. **B**, **C** Quantification of insoluble TDP-43 normalized by soluble TDP-43. Data are shown as mean ± SEM (*n* = 3). Note that MRPs and RBPs, which are classified into Group 1, decreased solubility both of TDP-43^∆NLS^-EGFP (**B**) and endogenous TDP-43 (**C**) but the gene products classified into Group 2 or 3 did not.

Furthermore, we also confirmed that the fluorescent-based screening results (Figs. 1 and 2) are well consistent with the results of TDP-43 insolubility assay (Fig. 3). The ALS/FTLD causative genes classified into Group 1 specifically decreased solubility of both TDP-43^∆NLS^-EGFP (Fig. 3B) and endogenous TDP-43 (Fig. 3C), supporting the notion that MRPs and RBPs are tightly involved in TDP-43 cytoplasmic aggregation.

### Liquid–liquid phase separation (LLPS) is involved in RBPs-induced TDP-43 aggregation

**Fig. 4:**
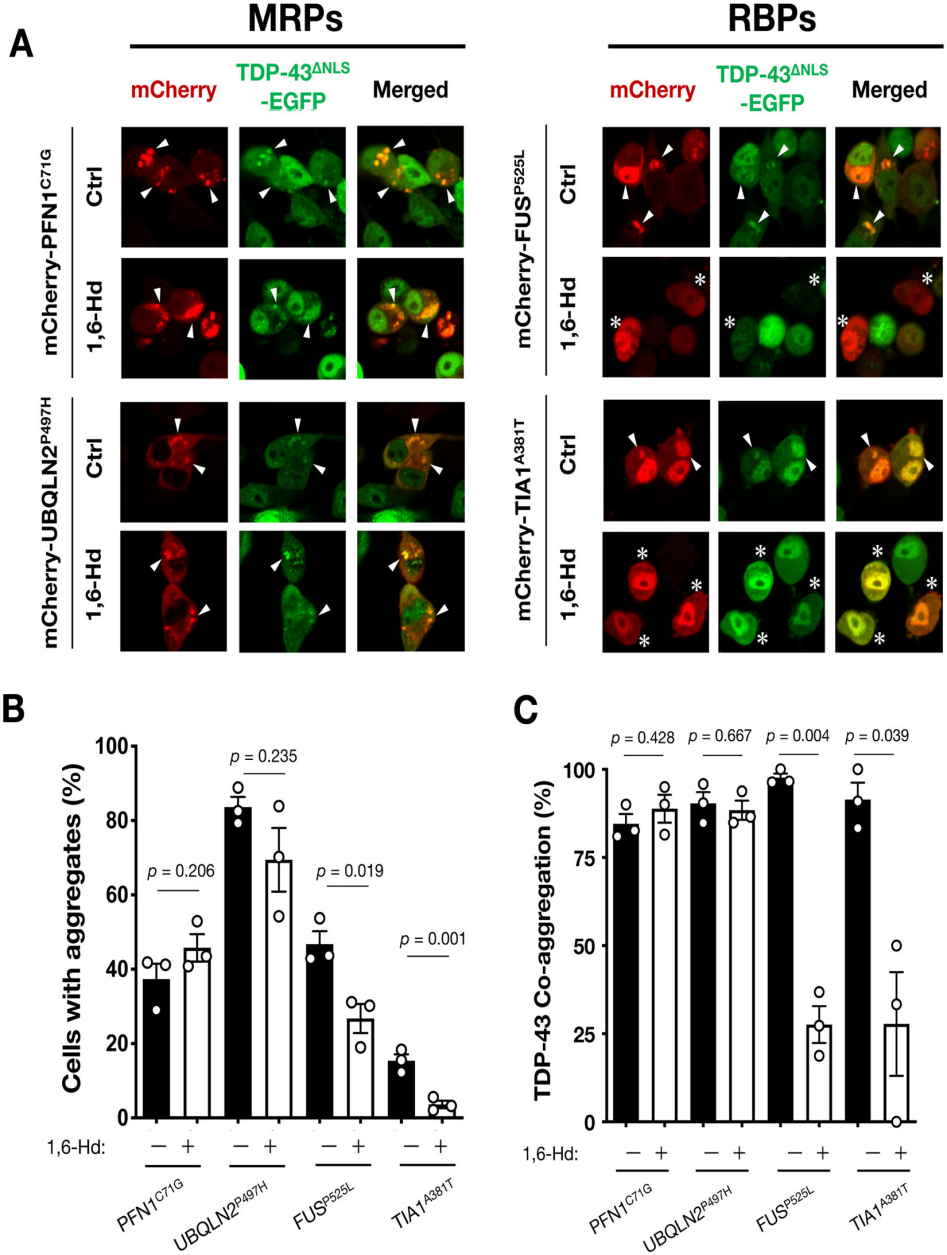
Liquid–liquid phase separation (LLPS) is involved in RBPs-induced TDP-43 co-aggregation. Administration of 1,6-hexanediol (1,6-Hd), an inhibitor of LLPS, dissociated TDP-43 co-aggregates with RBPs but not with MRPs in N2a cells expressing TDP-43^∆NLS^-EGFP. Representative images are shown in **A**. Both the ratio of the cells with TDP-43 aggregates (**B**) and TDP-43 co-aggregation (**C**) were reduced, specifically in the cells expressing RBPs with 1,6-Hd. Arrowheads indicate co-aggregates of the RBPs and MAPs with TDP-43^∆NLS^-EGFP. Whereas, asterisks indicate the cells without co-aggregates of the RBPs by the 1,6-Hd treatment. More than 50 cells in each condition were analyzed for the quantification. Data are expressed as mean ± SEM (*n* = 3).

Co-transfection of MRP (PFN1^C71G^) and RBP (FUS^P525L^) in N2a reporter cells stably expressing EGFP-TDP-43^∆NLS^ revealed that the aggregates induced by MRPs and RBPs were distinctly partitioned in cytoplasm (Supplementary Fig. S3). These results suggest that the mechanisms of MRPs- and RBPs-induced TDP-43 aggregation are independent of each other. Previous studies reported that FUS and TIA-1, representative RBPs classified into Group 1, form cytoplasmic aggregates through LLPS^24,25^. Thus, to investigate the mechanism of RBPs-induced aggregation, we treated the cells with 1,6-hexanediol (1,6-Hd), an inhibitor of LLPS, to assess the involvement of LLPS in the co-aggregation of RBPs and TDP-43. As shown in Fig. 4A, B, 1,6-Hd drastically dissociated the cytoplasmic aggregates of RBPs, mutant *FUS* and *TIA1*. Consistent with the decreased number of RBPs-induced aggregates, sequestration of TDP-43 into the aggregates was also significantly prevented (Fig. 4C). These observations suggest that LLPS drives the cytoplasmic co-aggregation of RBPs and TDP-43. In contrast, 1,6-Hd did not influence either the number of aggregates of MRPs (mutant *PFN1* and *UBQLN2*) or co-aggregates with TDP-43 (Fig. 4A–C), suggesting that a mechanism independent of LLPS drives the co-aggregation of MRPs and TDP-43.

### MRPs-induced TDP-43 co-aggregates are sensitive to nocodazole and co-localized with aggresomal markers

**Fig. 5:**
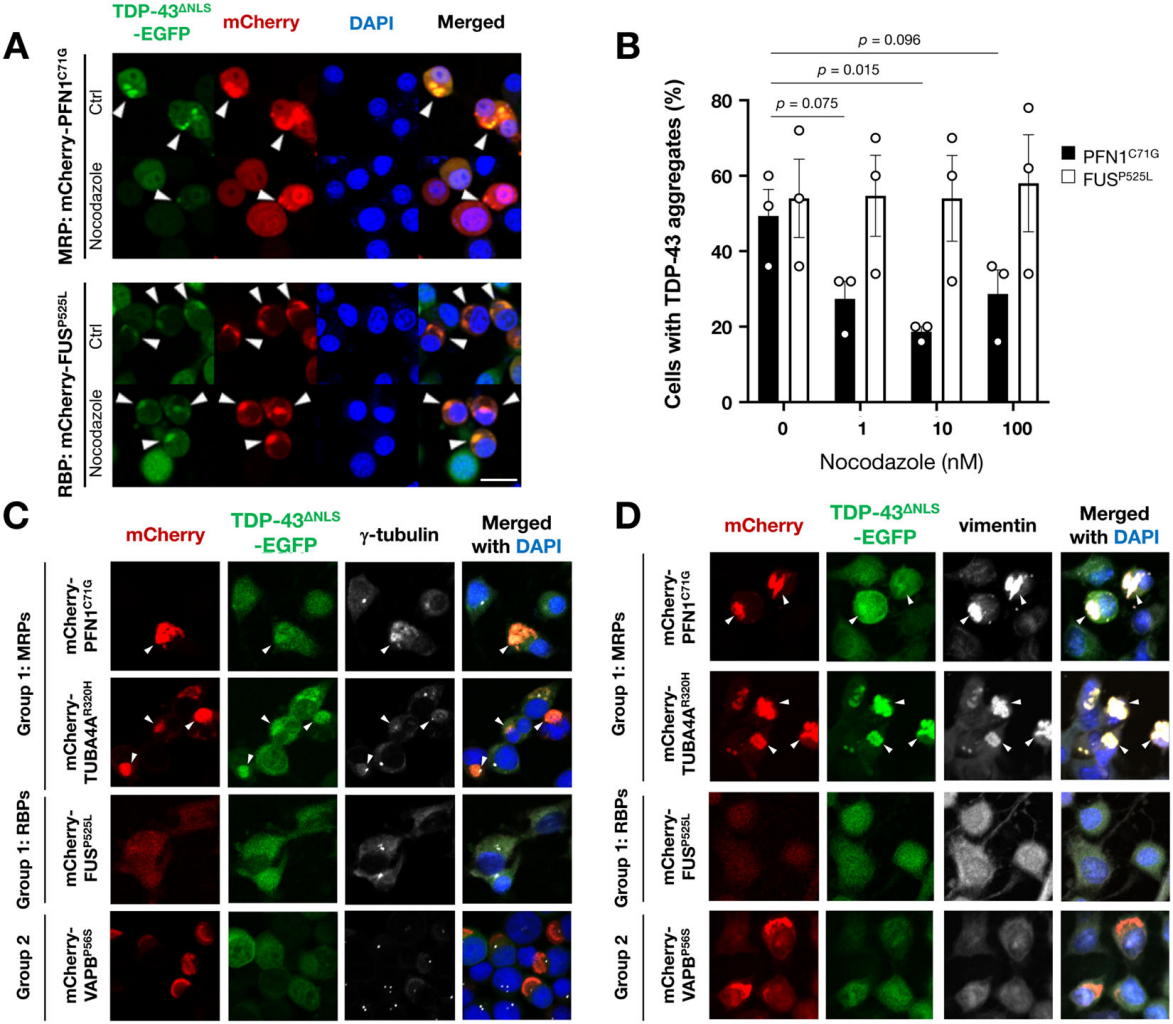
MRPs-induced TDP-43 aggregates are dependent on microtubules and co-localized with aggresome markers. **A, B** Nocodazole, a microtubule destabilizing reagent, dissociated TDP-43 co-aggregates with the MRP (PFN1) but not with the RBP (FUS). Arrowheads indicate co-aggregates of the MRP/RBP and TDP-43^∆NLS^-EGFP (**A**). The ratio of the cells with TDP-43 aggregates was quantified by counting more than 100 cells in each trial (**B**). Data are expressed as mean ± SEM (*n* = 3). **C**, **D** Representative immunofluorescence images showed that the MRPs (PFN1 and TUBA4A) induced TDP-43 co-aggregates were co-localized with the aggresomal markers γ-tubulin (**C**) and vimentin (**D**). Arrowheads indicate the co-localization of MRPs, TDP-43, and γ-tubulin in **C** or vimentin in **D**. Note that there were no aggregates in RBPs, represented by FUS, because the aggregates of RBPs were not observed in this immunocytochemical condition. Scale bars = 20 µm.

To examine whether the microtubules are essential for MRPs-induced TDP-43 aggregation, we then treated the cells with nocodazole, a microtubule destabilizing reagent. As expected, the nocodazole treatment substantially reduced the amount of co-aggregates of MRPs (PFN1^C71G^) and TDP-43 (Fig. 5A, B). Intriguingly, the co-aggregates of MRPs and TDP-43 were co-localized with the aggresomal markers γ-tubulin (Fig. 5C) and vimentin (Fig. 5D). An aggresome is a peri-nuclear compartment where misfolded proteins accumulate^26,27^. Once the cellular protein degradation system is overwhelmed, the misfolded proteins are accumulated into aggresomes through microtubules for a cellular defense. Therefore, our findings suggest that TDP-43 is sequestered into aggregates of MRPs during aggresome formation. Meanwhile, RBPs-induced TDP-43 co-aggregates (FUS^P525L^) were not affected by nocodazole (Fig. 5A) and did not co-localize with aggresomal markers (Fig. 5C, D), suggesting that MRPs and RBPs sequester TDP-43 into their aggregates through distinct mechanisms.

### Histone deacetylase 6 is associated with the co-aggregation of MRPs and TDP-43

**Fig. 6:**
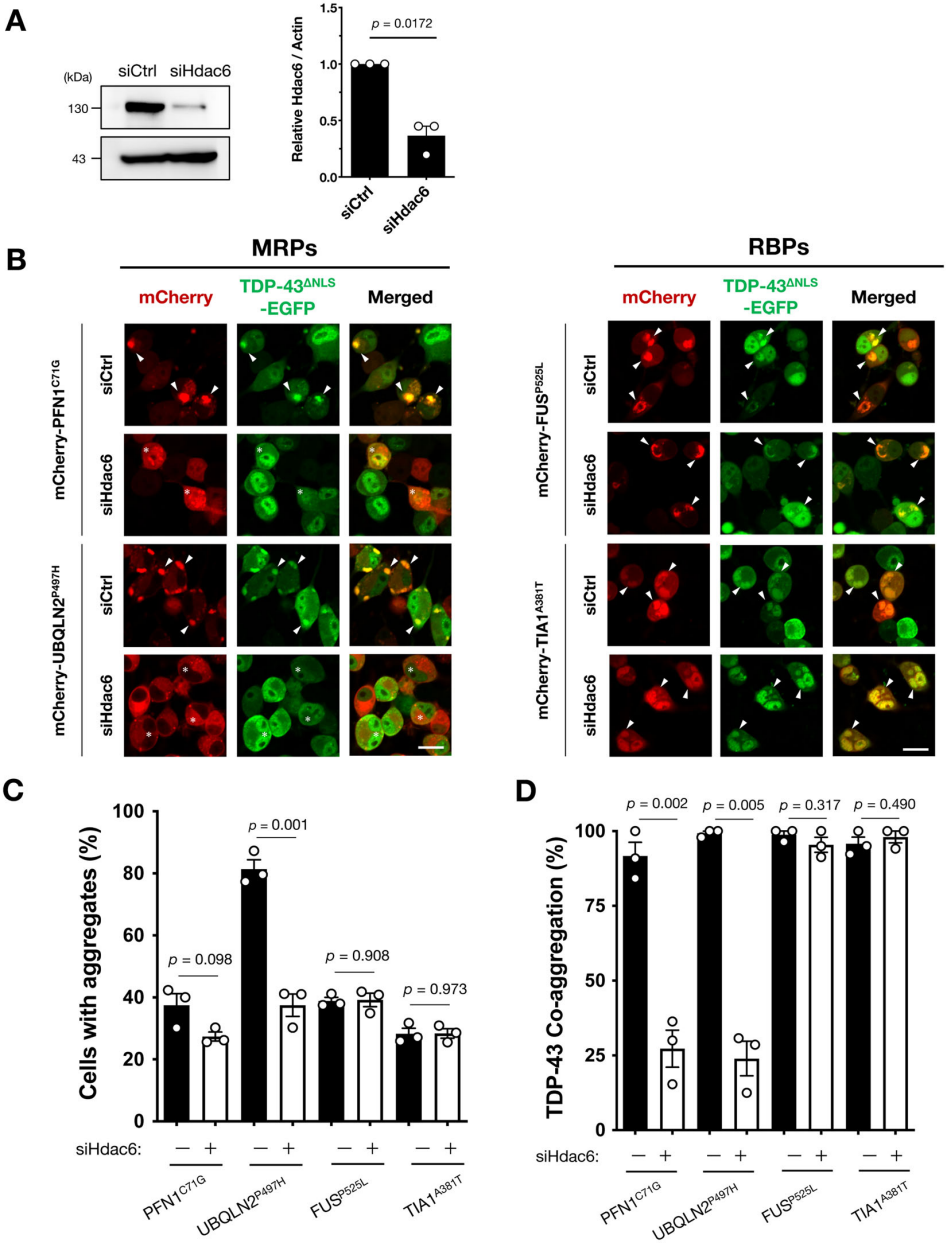
Histone deacetylase 6 (Hdac6) is involved in MRPs-induced TDP-43 co-aggregation. **A** Confirmation of Hdac6 knock-down in N2a cells by the treatment with siRNA (siHdac6). The expression levels relative to the control siRNA treated samples (siCtrl) were shown. **B**–**D** Hdac6 suppression specifically prevented MRPs-induced TDP-43 co-aggregation but not RBPs. Representative images are shown in **B**. Arrowheads indicate co-aggregates of the MRPs and RBPs with TDP-43^∆NLS^-EGFP. Whereas, asterisks indicate the cells without co-aggregates of the MRPs by Hdac6 suppression. The ratio of the cells with MRPs aggregates (**C**) and the ratio of MRPs and TDP-43 co-aggregation were substantially reduced, specifically by siHdac6 treatment (**D**). More than 50 cells in each condition were analyzed for the quantification. All the data are expressed as mean ± SEM (*n* = 3).

Histone deacetylase 6 (HDAC6) recognizes misfolded proteins and transports them to aggresomes through microtubules^28^. Previous reports showed that aggresomes are involved in the formation of inclusion bodies in the lesions of neurodegenerative diseases such as Huntington’s disease^29,30^. Therefore, we next examined whether the elimination of Hdac6 by siRNA affected TDP-43 co-aggregation with MRPs in N2a cells (Fig. 6A). As shown in Fig. 6B–D, Hdac6 knock-down prevented the aggregation of MRPs, as well as TDP-43 co-aggregation with MRPs. Interestingly, relatively small cytoplasmic aggregates of MRPs were still observed in the cells with Hdac6 knock-down (Fig. 6B, asterisks); however, TDP-43 was not co-localized with them. Since HDAC6 is crucial for the transport of misfolded proteins to aggresomes^28,29^, this observation might suggest that TDP-43 is sequestered during compartmentalization. On the other hand, the Hdac6 knock-down did not affect RBPs-induced TDP-43 co-aggregation, indicating that Hdac6 was not involved in the TDP-43 sequestration into the LLPS-induced aggregates.

### Different characteristics of TDP-43 inclusions in sporadic ALS suggest multiple pathways form TDP-43 pathology

**Fig. 7:**
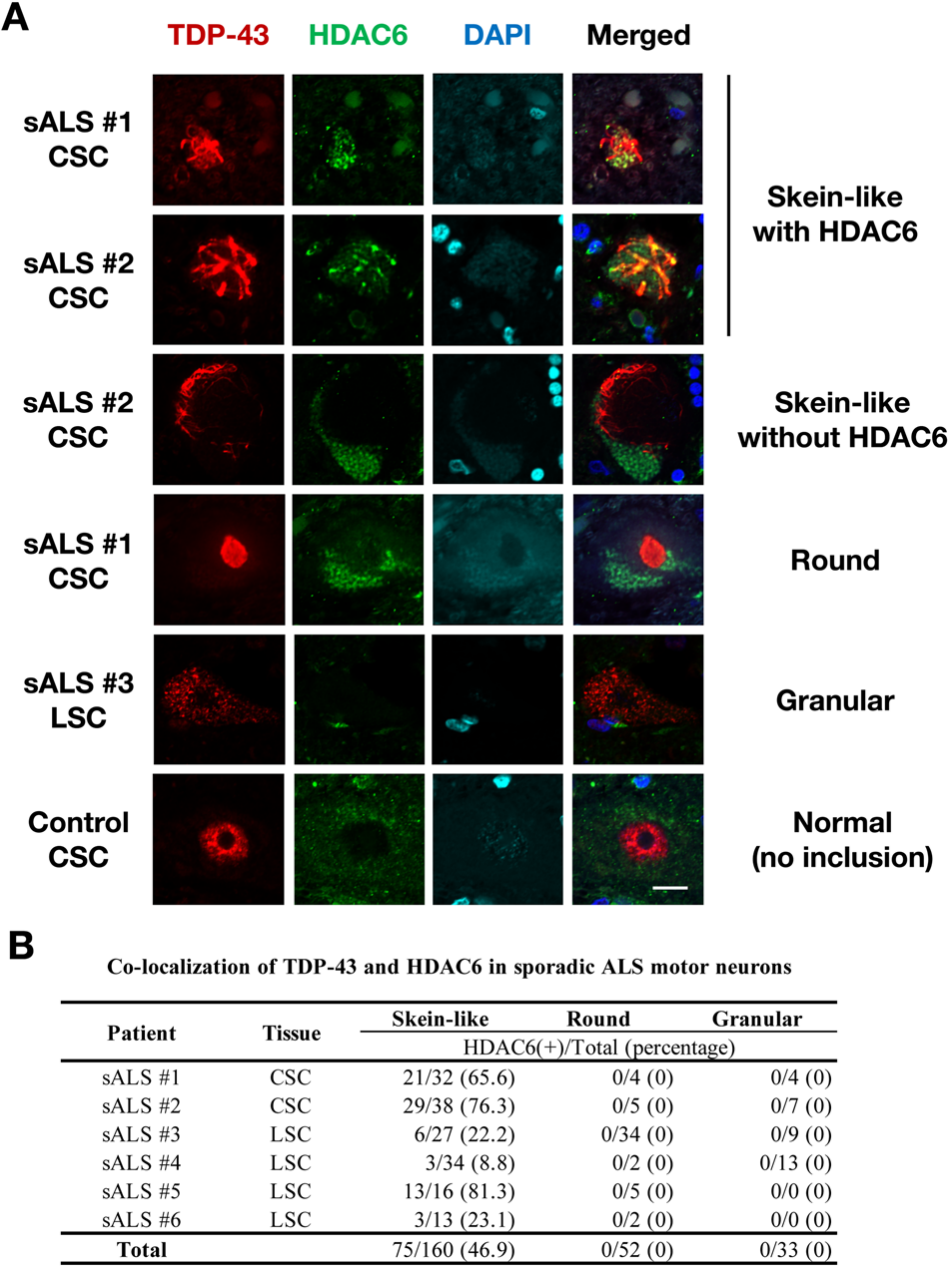
A part of skein-like cytoplasmic inclusion of TDP-43 co-localized with HDAC6 in the spinal motor neurons of sporadic ALS patients. **A** Representative immunofluorescent images of the spinal cord sections of sporadic ALS patients stained with TDP-43, HDAC6, and DAPI demonstrate various TDP-43-immunopositive inclusions. Note that only the skein-like inclusions of TDP-43 co-localized with HDAC6. CSC Cervical Spinal Cord, LSC Lumbar Spinal Cord. Scale bar = 20 µm. **B** A summary of the quantification of co-localization of TDP-43 and HDAC6 in sporadic ALS motor neurons.

The results of TDP-43 co-aggregation obtained by our screening system are based on the overexpression of the inherited ALS/FTLD causative genes. However, it is unclear whether the mechanisms for the formation of TDP-43 aggregation observed in our study are involved in the TDP-43 pathology of sporadic ALS. To examine the involvement of the aggresome pathway in TDP-43 pathology, we examined the co-localization of TDP-43 and HDAC6, an aggresomal marker, in spinal motor neurons of sporadic ALS patients. We found that approximately half of TDP-43 skein-like inclusions were co-localized with HDAC6 (75/160, 46.9% of total skein-like inclusions) (Fig. 7A, B). It should be noted that no co-localization of TDP-43 and HDAC6 was observed in round (0/52) or granular (0/33) TDP-43 inclusions. These observations support the hypothesis that TDP-43 skein-like inclusions are, at least partially, formed by a similar mechanism to aggresome formation. Importantly, both the HDAC6-positive and HDAC6-negative inclusions were found in every sporadic ALS patient, suggesting that the mechanisms of TDP-43 aggregation vary in each of the neurons. In conclusion, the different characteristics between skein-like and other types of TDP-43 inclusions suggest that TDP-43 pathology is developed via multiple complexed mechanisms, likely including both aggresome formation and LLPS, in sporadic ALS.

## Discussion

In this study, we demonstrated that: (i) at least two distinct intracellular pathways were involved in TDP-43 cytoplasmic aggregation, including aggresome formation and LLPS; (ii) the two pathways were independent of each other; and (iii) in spinal motor neurons of sporadic ALS, both HDAC6-positive and HDAC6-negative aggregates were found in the same ALS patient. Therefore, to the best of our knowledge, we are the first to confirm that HDAC6-dependent aggresome formation is partially involved in skein-like TDP-43 inclusion of sporadic ALS.

Our study revealed that over 70% of the examined inherited ALS/FTLD causative 19 genes induced TDP-43 co-aggregation in cultured neuronal cells and the fluorescent-based observation of TDP-43 co-aggregates was well consistent with the TDP-43 insolubility examined by immunoblotting. Our screening system enabled us to assess whether any genes of interest induce cytoplasmic TDP-43 aggregation by a simple transfection-based screening. Moreover, our screening system has been proved useful for investigating the mechanism of TDP-43 aggregation. Indeed, as described above, we identified two distinct mechanisms involved in TDP-43 aggregation by using this system (i.e., aggresome formation and LLPS). Our screening system will also be useful for identifying compounds that prevent cytoplasmic TDP-43 aggregation, which may contribute to a novel therapeutic strategy for ALS/FTLD.

Although our screening system generally well recapitulated cytoplasmic TDP-43 aggregation in ALS/FTLD, our results were partially inconsistent with some previous reports on the pathology of inherited ALS^18,19^. Notably, we did not find any evidence of TDP-43 aggregation in the neuronal cells expressing the inherited ALS/FTLD causative genes related to protein degradation (e.g., *VCP*, *UBQLN2*, *SQSTM1*, and *TBK1*), although the previous studies reported TDP-43 pathology in inherited ALS patients with these mutations^18^. Moreover, the co-localization of TDP-43 and the inherited ALS/FTLD causative gene products are still controversial even though TDP-43 pathology is widely observed in the patients with ALS. For example, some studies have suggested the co-localization of TDP-43 with TIA-1^31^ or FUS^32^ but other studies do not^33,34^. One possible interpretation of this inconsistency is an experimental limitation of our screening system based on the overexpression of the inherited ALS/FTLD causative genes with a short incubation time before evaluation. Therefore, a careful interpretation is required to compare our results with the inherited ALS/FTLD neuropathology data.

The co-aggregation of TDP-43 and RBPs was sensitive to 1,6-Hd, suggesting that LLPS drives cytoplasmic RBP-induced aggregation. Nucleotides, enriched in nuclei, prevent aggregation driven by LLPS^35,36^. Indeed, the previous study demonstrated that an artificially designed RNA, which specifically binds to TDP-43, prevents intracellular aggregation of TDP-43 triggered by light-driven forced multimerization of TDP-43 in the cytoplasm^17^. This mechanism could explain our observations that RBPs only aggregated with TDP-43 when they leaked from nuclei into the cytoplasm. On the other hand, the MRPs-induced co-aggregation of TDP-43 was dependent on aggresome formation. The role of aggresomes in the accumulation of misfolded proteins has been pointed out in various neurodegenerative diseases^37^. ALS-linked PFN1 mutant proteins that aggregated in the cytoplasm were dependent on microtubules, which was similar to our observations^38^. Intriguingly, these two pathways of TDP-43 aggregation are not complementary and are independent of each other. Inhibiting LLPS prevented RBPs-mediated TDP-43 aggregation, but did not affect MRPs-induced TDP-43 aggregation. Conversely, HDAC6 suppression and microtubular destabilization only affected MRPs-induced TDP-43 aggregation. All these observations indicate that targeting the intracellular mechanism specific to each pathway is key to preventing TDP-43 accumulation. Nonetheless, there is still a possibility that other independent pathways are also involved in cytoplasmic TDP-43 aggregation. Further investigation into the potential mechanisms of TDP-43 accumulation is required.

HDAC6 is a key molecule in aggresome formation. HDAC6 facilitates the transport of misfolded proteins by connecting microtubules and ubiquitinated proteins^28,37^. The involvement of HDAC6 has been reported in various neurodegenerative diseases such as Huntington’s disease^29^, Parkinson’s disease^28^, and Alzheimer’s disease^30^. In ALS, it was reported that deletion of *Hdac6* ameliorates the disease progression in SOD1-ALS mice^39^ and TDP-43 deficiency drastically reduces HDAC6 expression in cultured cells^40^. Moreover, HDAC6 inhibition reversed axonal transports, which are defective in iPS-derived motor neurons from ALS patients with FUS mutation^41^. These observations suggest the possibility that HDAC6 is also involved in the ALS pathology similar to other neurodegenerative diseases and that it may be a therapeutic target for ALS through restoring axonal transport. In addition to this, our results strengthen the possibility that inhibition of HDAC6 is a potential therapeutic target for ALS by providing a novel mechanism for preventing microtubule-dependent TDP-43 pathology. HDAC6 inhibition is probably effective for MRPs-linked inherited ALS and partially to sporadic ALS.

Since most of the ALS cases are sporadic, it is important to identify the mechanism responsible for TDP-43 aggregation in the sporadic ALS. Recent reports showed that cytoplasmic TDP-43 was sequestered into aggresome dependent on microtubules in fibroblasts derived from sporadic ALS patients^42^. Consistent with the cited study, we found that TDP-43 was sequestered into aggregates of MRPs in N2a cells and approximately half of TDP-43 skein-like inclusions in sporadic ALS patients were co-localized with HDAC6, an aggresomal marker. Our results suggest that the HDAC6-dependent aggresome pathway may be involved in the formation of skein-like inclusion in ALS/FTLD. However, we also found that both HDAC6-positive and HDAC6-negative skein-like inclusions were observed in the same sporadic ALS patient, as shown in Fig. 7B. In addition, the round and granular inclusions, which are not co-localized with HDAC6, were also found in the same sporadic ALS patient. These observations suggest that the mechanisms responsible for TDP-43 aggregation are different from each other among the remaining motor neurons in the one sporadic ALS patient and that the morphology of TDP-43 inclusions may reflect the corresponding intracellular mechanism. Further studies are needed to determine the mechanism responsible for each type of cytoplasmic TDP-43 aggregation in motor neurons. We found that the two distinct pathways, aggresome formation and LLPS, are independently involved in the formation of cytoplasmic TDP-43 inclusions in ALS. Although it is unclear whether each type of cytoplasmic TDP-43 aggregates provokes toxicities to neurons, our findings, together with the other studies, suggest the therapeutic potential of HDAC6 through the several lines of mechanisms in ALS/FTLD.

## Supplementary information

Supplementary Information

Supplementary Figure S1

Supplementary Figure S2

Supplementary Figure S3

Supplementary Table S1
