# Supplementary material for: Aggresome formation and liquid–liquid phase separation independently induce cytoplasmic aggregation of TAR DNA-binding protein 43
Source: Cell Death Dis. 2020 Oct 23;11(10):909. doi: 10.1038/s41419-020-03116-2 (PMC7585435; doi:10.1038/s41419-020-03116-2)
Supplement: Supplementary file 1 — Supplementary Information [file 41419_2020_3116_MOESM1_ESM.docx]

**Supplementary Information**

**Aggresome Formation and Liquid-liquid Phase Separation Independently Induce Cytoplasmic Aggregation of TAR DNA-binding protein 43**

Seiji Watanabe, Hidekazu Inami, Kotaro Oiwa, Yuri Murata, Shohei Sakai, Okiru Komine, Akira Sobue, Yohei Iguchi, Masahisa Katsuno, and Koji Yamanaka

**Supplementary Figure Legends**

**Supplementary Figure S1. Representative images of TDP-43^∆NLS^-EGFP co-aggregation screening**

The screening results are summarized in Table 1. Scale bars = 20 µm.

**Supplementary Figure S2. Sequestration of nuclear TDP-43^WT^-EGFP into the aggregates of ALS/FTLD-causative genes classified as Group 1**

In addition to PFN1, UBQLN2, FUS and TIA1, all the other genes classified into Group 1 (TUBA4A, CHMP2B, TAF15, MATR3) also induced sequestration of TDP-43^WT^-EGFP into their cytoplasmic aggregates. Arrows represent typical co-localization of the gene products labeled by mCherry (red) and TDP-43^WT^-EGFP (green). Scale bar = 20 µm.

**Supplementary Figure S3. Aggregates formed by PFN1^C71G^ (MRP) was distinctly partitioned from FUS^P525L^ in N2a cells.**

The N2a cells were co-transfected with mCherry-PFN1^C71G^ and 3×FLAG-FUS^P525L^ mammalian expression plasmids. Note that PFN1^C71G^ and FUS^P525L^ were completely separated in the cells and that TDP-43^∆NLS^-EGFP was colocalized with PFN1^C71G^ rather than FUS^P525L^ in this condition. Scale bar = 10 µm.

**Supplementary Table S1. Clinical details of the patients**

**Supplementary Experimental Procedure**

***Immunofluorescence of PFN1 and FUS co-transfected N2a cells***

The N2a･TDP-43^∆NLS^- EGFP were seeded at 5.0 × 10^4^ /well on poly-D-lysine coated 4 well slide chamber (Thermo). On the next day, the cells were transfected with 0.4 µg/well pmCherry-PFN1^C71G^ and pF5K/3×FLAG-FUS^P525L 1^ using Lipofectamine 2000 according to the manufacturer’s protocol. After 6 h of transfection, the medium was changed to the differentiation medium, and the cells were incubated for 48 h. The cells were fixed with 4 % paraformaldehyde in phosphate buffer at room temperature for 20 min. After three times wash with TBS, the cells were immunostained as described in Immunofluorescence method described in the main text.

**Supplementary Reference**

1 Tsuiji, H. et al. Spliceosome integrity is defective in the motor neuron diseases ALS and SMA. *EMBO Mol. Med.* **5**, 221-234 (2013).
