# Supplementary figures and images for: Aggresome formation and liquid–liquid phase separation independently induce cytoplasmic aggregation of TAR DNA-binding protein 43
Source: Cell Death Dis. 2020 Oct 23;11(10):909. doi: 10.1038/s41419-020-03116-2 (PMC7585435; doi:10.1038/s41419-020-03116-2)

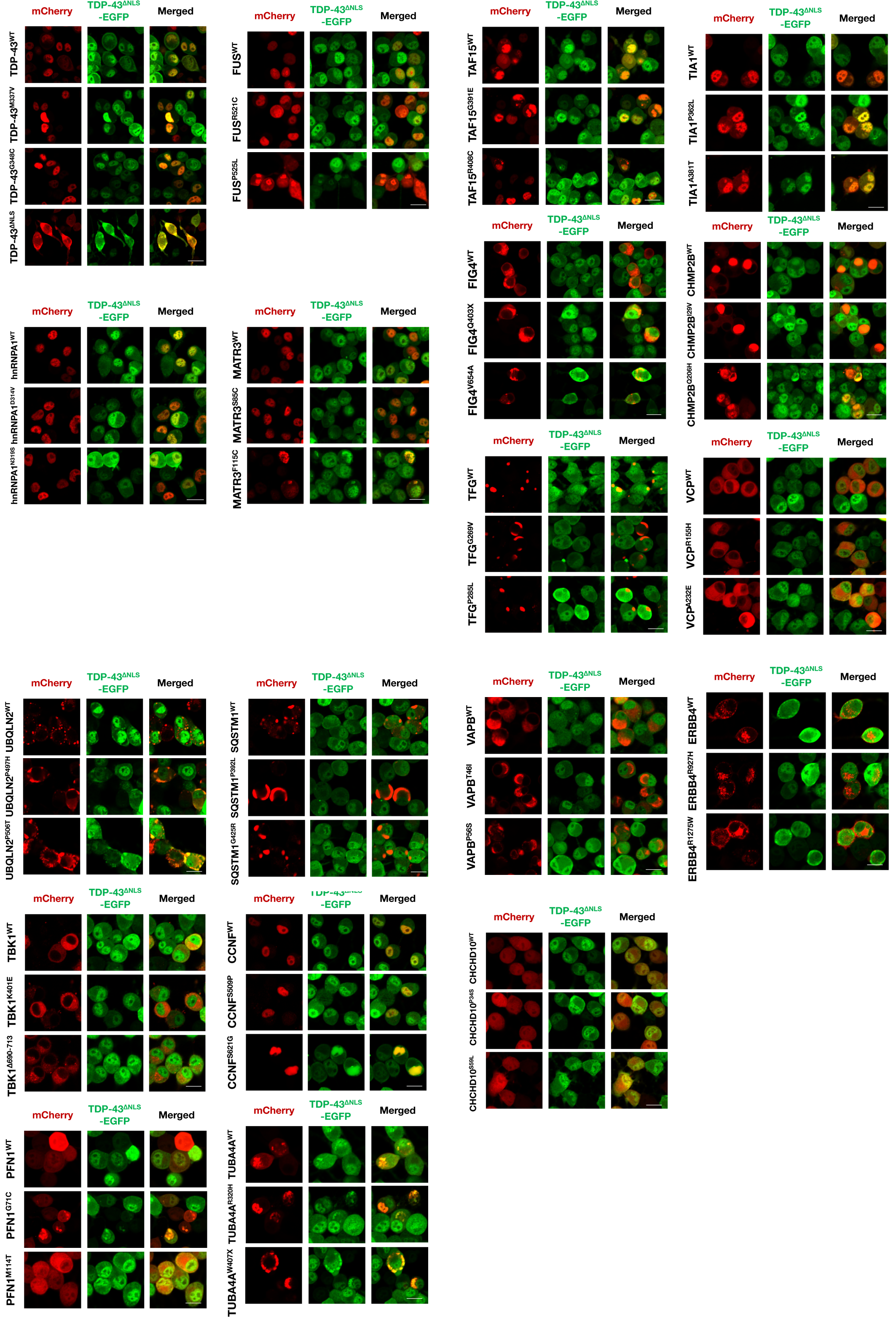

Supplement: Supplementary file 2 — Supplementary Figure S1 [file 41419_2020_3116_MOESM2_ESM.tif]

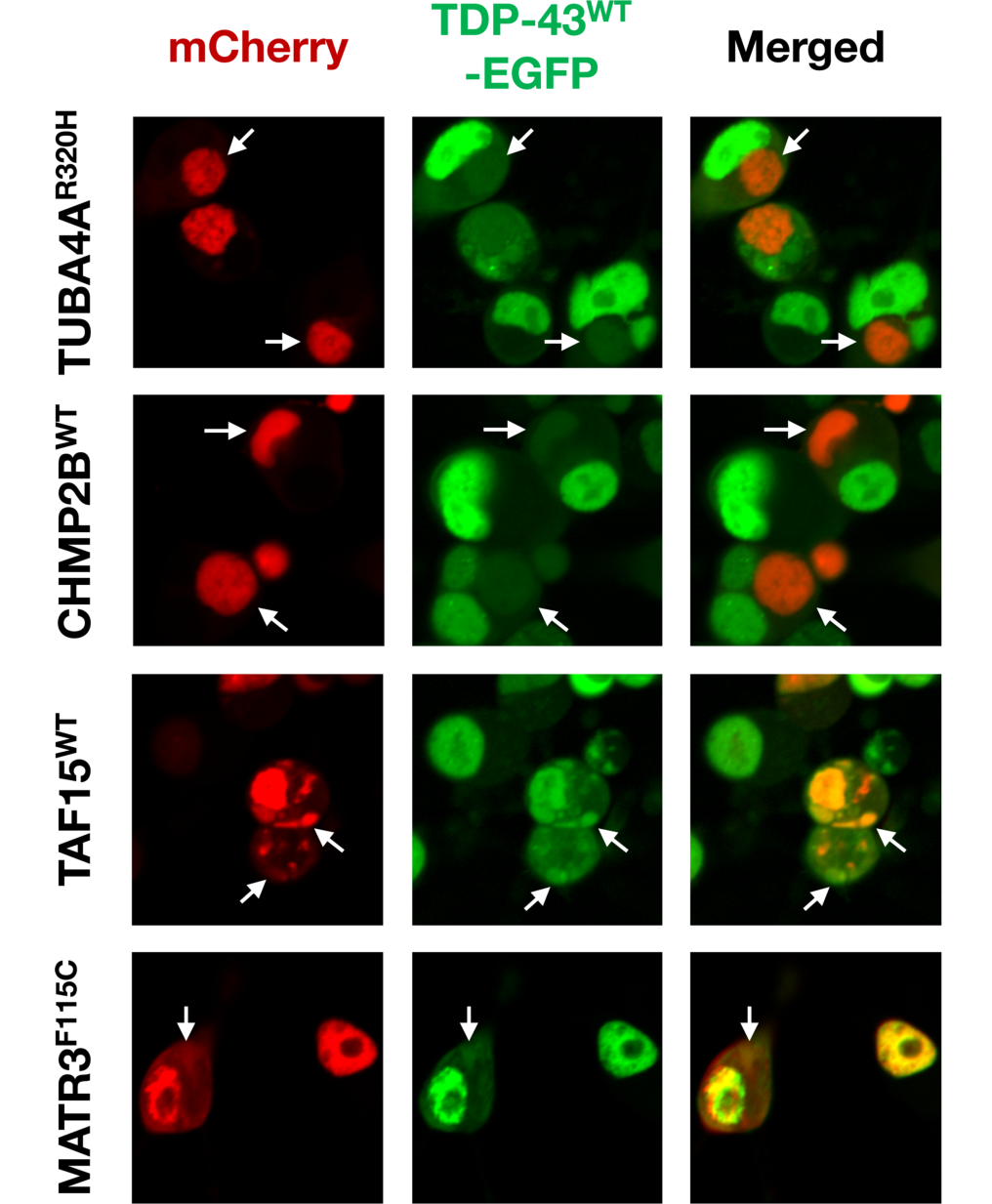

Supplement: Supplementary file 3 — Supplementary Figure S2 [file 41419_2020_3116_MOESM3_ESM.tif]

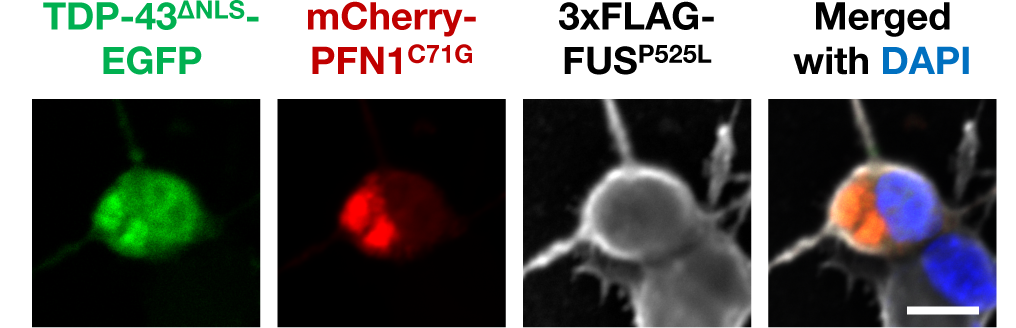

Supplement: Supplementary file 4 — Supplementary Figure S3 [file 41419_2020_3116_MOESM4_ESM.tif]
